# Surgical experience of extracorporeal membrane oxygenation for neonates with severe respiratory failure

**DOI:** 10.1186/s12893-023-02094-4

**Published:** 2023-07-06

**Authors:** Qi-Liang Zhang, Xiu-Hua Chen, Si-Jia Zhou, Hua Cao, Qiang Chen

**Affiliations:** Department of Cardiac Surgery, Fujian Children’s Hospital (Fujian Branch of Shanghai Children’s Medical Center), College of Clinical Medicine for Obstetrics & Gynecology and Pediatrics, Fujian Medical University, Fuzhou, China

**Keywords:** Neonates, Respiratory failure, ECMO, Cannulation of the internal jugular vein and carotid artery, Surgical experience

## Abstract

**Objective:**

Extracorporeal membrane oxygenation (ECMO) has been increasingly used for severe neonatal respiratory failure refractory to conventional treatments. This paper summarizes our operation experience of neonatal ECMO via cannulation of the internal jugular vein and carotid artery.

**Methods:**

The clinical data of 12 neonates with severe respiratory failure who underwent ECMO via the internal jugular vein and carotid artery in our hospital from January 2021 to October 2022 were collected.

**Results:**

All neonates were successfully operated on. The size of arterial intubation was 8 F, and the size of venous intubation was 10 F. The operation time was 29 (22–40) minutes. ECMO was successfully removed in 8 neonates. Surgeons successfully reconstructed the internal jugular vein and carotid artery of these neonates. Arterial blood flow was unobstructed in 5 patients, mild stenosis was present in 2 patients, and moderate stenosis was present in 1 patient. Venous blood flow was unobstructed in 6 patients, mild stenosis was present in 1 patient, and moderate stenosis was present in 1 patient. The complications were as follows: 1 case had poor neck incision healing after ECMO removal. No complications, such as incisional bleeding, incisional infection, catheter-related blood infection, cannulation accidentally pulling away, vascular laceration, thrombosis, cerebral haemorrhage, cerebral infarction, or haemolysis, occurred in any of the patients.

**Conclusion:**

Cannulation of the internal jugular vein and carotid artery can quickly establish effective ECMO access for neonates with severe respiratory failure. Careful, skilled and delicate operation was essential. In addition, during the cannulation process, we should pay special attention to the position of cannulation, firm fixation and strict aseptic operation.

## Introduction

Respiratory failure is the most common cause of neonatal death. Common causes of neonatal respiratory failure include meconium aspiration syndrome, persistent pulmonary hypertension, congenital diaphragmatic hernia and acute respiratory distress syndrome [1–3]. With the continuous progress of respiratory support technologies, including pulmonary surfactants, high-frequency oscillatory ventilation and nitric oxide, most patients can be saved, but there are still some neonates with severe respiratory failure who need extracorporeal membrane oxygenation (ECMO) support [4, 5].

ECMO drains blood from the body to the outside through arteriovenous intubation, and then oxygenated blood is injected into the body via a pump after artificial membrane lcrng oxygenation to maintain the blood supply and oxygen supply to various organs of the body. ECMO provides respiratory and cardiac support to patients with severe cardiopulmonary failure, allowing the heart and lung to fully rest, and allowing valuable time for further treatment and recovery of cardiopulmonary function [6]. ECMO is a high-risk procedure that needs to be performed quickly and effectively in an emergency. The first step of ECMO is to establish effective circulation access, and neonates are usually intubated by the internal jugular vein and carotid artery [7]. Due to the small blood vessels and small blood volume of neonates, the proper position of cannulation is necessary If the position of cannulation is unsatisfactory, the ECMO will be affected, which will bring potential risks. Meanwhile, the repeated adjustment of the position of cannulation will not only increase the risk of infection but also damage the blood vessel. Therefore, skilled, rapid and accurate cannulation of the internal jugular vein and carotid artery is essential to ensure stable ECMO flow and reduce the number of cannulation adjustments. At present, there is no uniform standard for neonatal ECMO cannulation. On the basis of summarizing domestic and foreign ECMO cannulation experience, we improved the cannulation technology. This paper summarizes the surgical experience of neonatal ECMO cannulation at our centre.

## Methods

### Patients

Twelve neonates with severe respiratory failure who underwent ECMO via the internal jugular vein and carotid artery in our hospital from January 2021 to October 2022 were enrolled. There were 9 males and 3 females with an age of 2 days (1–25 days) and a body weight of 3.4 kg (2.8–4.8) kg. The primary disease was severe pneumonia in 2 cases, persistent pulmonary hypertension combined with acute respiratory distress syndrome in 8 cases, and congenital diaphragmatic hernia in 2 cases.

### Team of ECMO

All patients received cannulation of ECMO bedside in the intensive care unit of cardiac surgery in our hospital. Two paediatric cardiac surgeons performed the cannulation of ECMO (the primary surgeon had more than 10 years of paediatric cardiac surgery experience). One physician and one nurse of the intensive care unit were responsible for monitoring the vital signs and ventilator status of the patients during intubation. One cardiac anaesthesiologist was responsible for the anaesthesia and intubation of the deep vein and arterial blood pressure before ECMO. One extracorporeal circulation perfusionist was responsible for the installation and work of ECMO.

### Method of anaesthesia

All patients were intubated and their breathing assisted with a ventilator. All patients were maintained under anaesthesia via midazolam 0.3 mg/kg, fentanyl 10–30 μg/kg and rocuronium 0.6 mg/kg.

### Technology of ECMO intubation

**Fig. 1:**
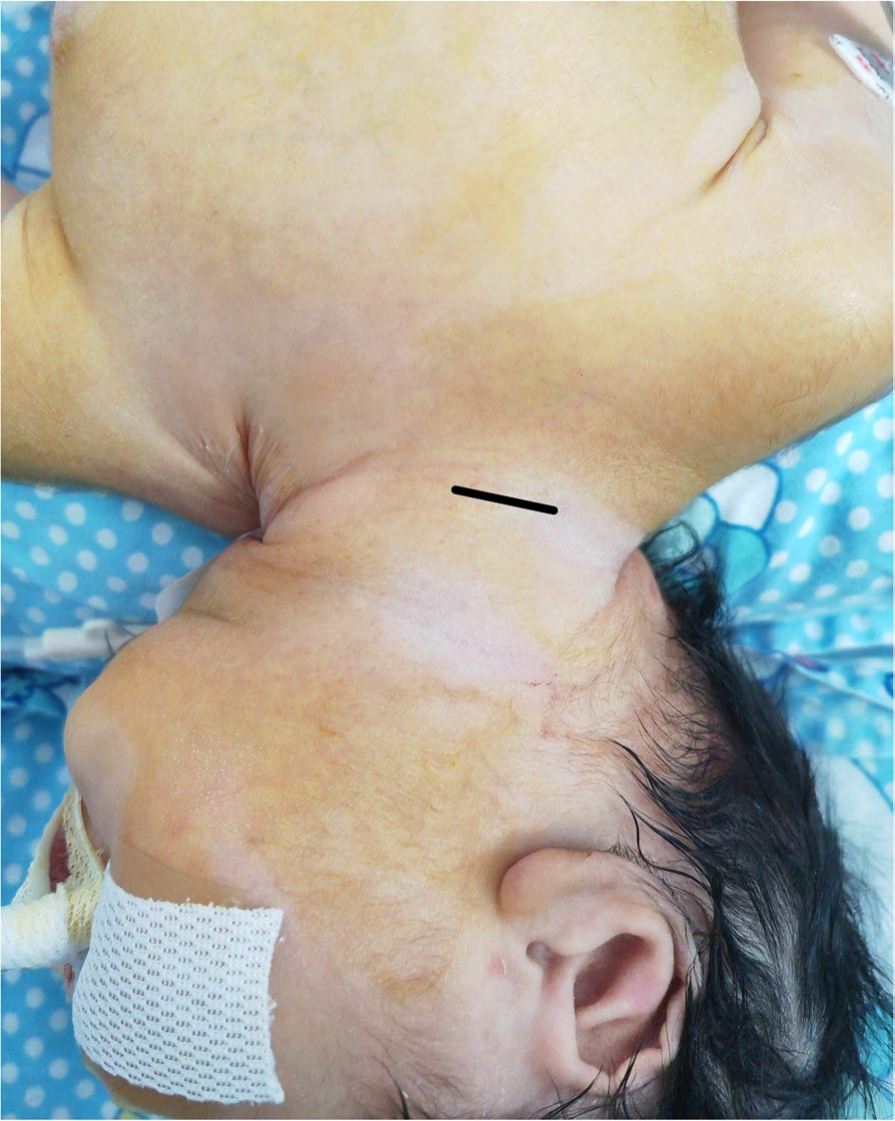
An incision of approximately 2 cm was made along the transverse line of the neck at 1–2 cm above the right supraclavicular fossa

**Fig. 2:**
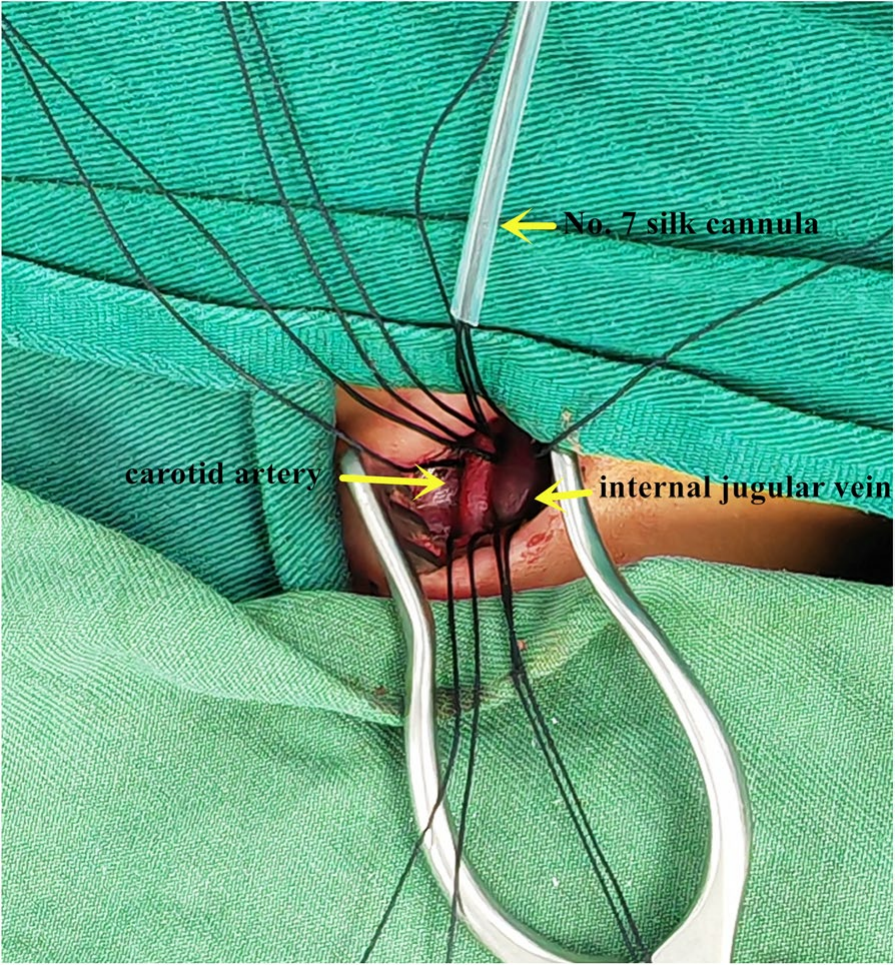
The internal jugular vein and the carotid artery were fully dissociated, and No. 7 silk was placed on the proximal and distal ends of the internal jugular vein and the carotid artery, respectively

**Fig. 3:**
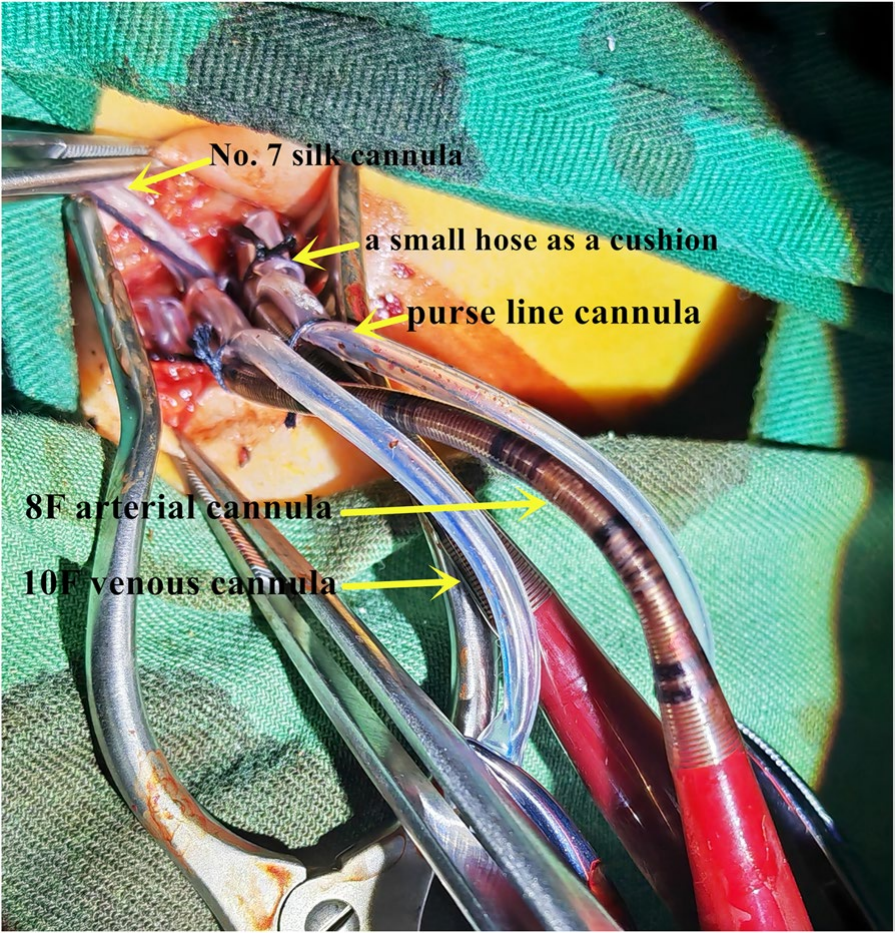
Intubation of the internal jugular vein and the carotid artery and fixation of cannulation

1. The patients were placed in a supine position, and the head was tilted back by padding the shoulders with cotton and turning to the left to fully expose the right neck.
2. An incision of approximately 2 cm was made along the transverse line of the neck at 1–2 cm above the right supraclavicular fossa (Fig. 1).
3. The superficial fascia was dissected to expose the leading edge of the sternocleidomastoid muscle. The sternocleidomastoid muscle was bluntly separated to the right to expose the carotid sheath.
4. The carotid arteries and veins were exposed by opening the carotid sheath.
5. The internal jugular vein and the carotid artery were fully dissociated, and No. 7 silk was placed on the proximal and distal ends of the internal jugular vein and the carotid artery, respectively (Fig. 2).
6. The 5–0 Prolene line was used to suture the purse on the surface of the carotid artery and vein, and the cannula was put on reserve.
7. The distal end of the carotid artery was blocked by blocking forceps, and then the proximal end was blocked.
8. After cutting open the artery in the purse, the 8F arterial cannula was inserted. At the same time, the proximal end of the heart-blocking forceps was opened, and then we inserted the arterial cannula and tightened the purse line.
9. The arterial cannula and the purse-line cannula were fixed with No. 7 silk. The distal ends of the heart-blocking forceps were opened.
10. An indwelling No. A 7 silk cannula secured the arterial cannula. Using a small hose as a cushion, the indwelling No. 7 silk thread ligated the distal end of the heart artery to prevent bleeding and secure the proximal end of the heart arterial cannula.
11. After the intubation was fixed, the core of cannulation was pulled out, and the ECMO artery and arterial cannula were connected.
12. The proximal end of the internal jugular vein was blocked by blocking forceps, and then the distal end was blocked.
13. After cutting open the vein in the purse, the 10F venous cannula was inserted. At the same time, the proximal end of the heart-blocking forceps was opened, and then we inserted the venous cannula and tightened the purse line.
14. The venous cannula and the purse-line cannula were fixed with No. 7 silk. The distal ends of the heart-blocking forceps were opened.
15. Using a small hose as a cushion, the indwelling No. 7 silk thread ligated the distal end of the heart vein to prevent bleeding and secure the proximal end of the heart venous cannula (Fig. 3).
16. After the intubation was fixed, the core of cannulation was pulled out, and the ECMO vein and venous cannula were connected.

### The intubation position

The transfer of ECMO started after the cannulation of ECMO was established successfully. We confirmed the cannulation location via bedside ultrasound examination. The ideal position of arterial cannulation was the tip of cannulation at the innominate artery entering the aortic arch, and the ideal position of venous cannulation was the tip of the cannulation at the inferior vena cava entering the right atrium. If the position was not satisfactory, we adjusted the intubation depth again. After confirming that the position and flow were satisfactory and without bleeding, we sutured the skin incision. The arterial cannulation and venous cannulation were sutured and fixed on the skin with silk.

### Technology of drawing out ECMO cannulation

**Fig. 4:**
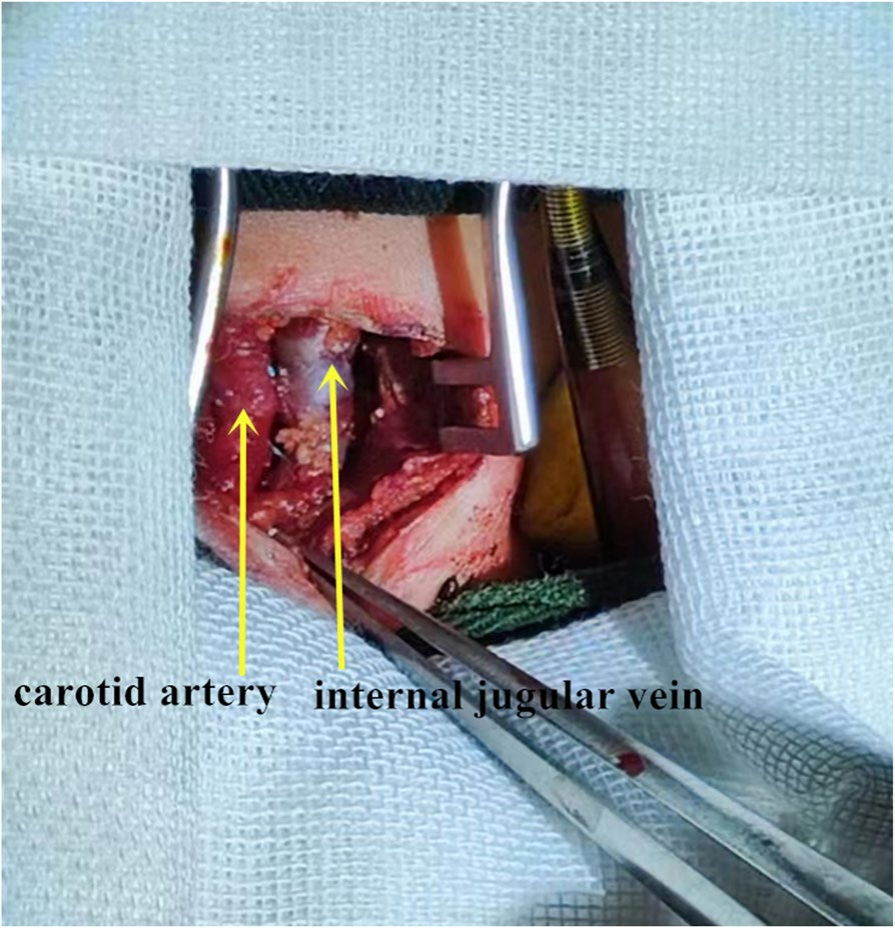
The internal jugular vein and the carotid artery incision were sutured with an 8–0 Prolene line

1. The anaesthesia process and the position of the patients were the same as the intubation process.
2. After disinfection, we cut the incision suture to expose the neck vessels and cannulation. We shut down ECMO and clamped the cannulation.
3. We cut the silk of fixed arteries and veins at the proximal end of the heart and cut the silk of ligating arteries and veins at the distal end of the heart.
4. Cannulation of the internal jugular vein was drawn out first. After loosening the cannula of the venous purse, we removed the cannulation of the internal jugular vein and tightened the purse line to prevent bleeding.
5. The same procedure was used to remove the cannulation of the carotid artery.
6. Occlusion forceps were used to block the proximal and distal ends of the internal jugular vein, and the purse line was removed.
7. After detecting the patency of the vessels in the proximal and distal ends, we sutured the closed vein incision with an 8–0 Prolene line. Then, we sutured the artery incision by the same method (Fig. 4).
8. After the wound was detected without bleeding, the incision was washed with hydrogen peroxide and normal saline successively.
9. The incision was closed with intermittent sutures, and a rubber drainage strip was indwelled.

## Results

All patients were successfully cannulated with 8F arterial cannulation (Medtronics company, America) and 10F cannulation (Medtronics company, America). The depth of cannulation of the carotid artery was 3 cm in 9 cases and 3.5 cm in 3 cases. The depth of cannulation of the internal jugular vein was 6.5 cm in 1 case, 7 cm in 8 cases and 7.5 cm in 3 cases. The operation time was 29 (22–40) minutes.

**Fig. 5:**
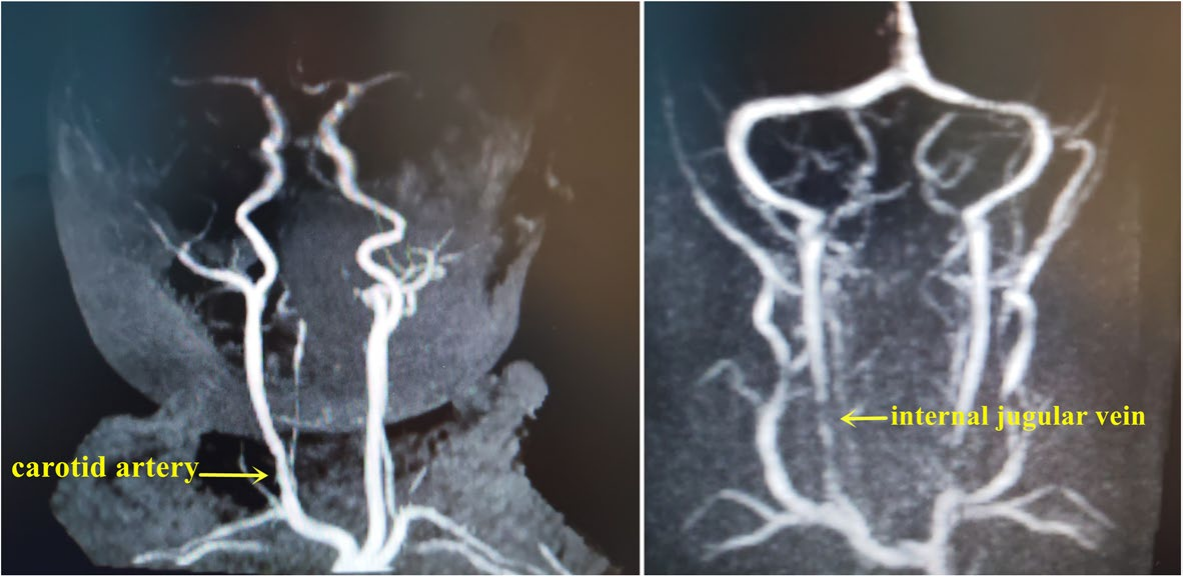
Postoperative MRA showed that the blood flow of the reconstructed internal jugular vein and the carotid artery were smooth

ECMO was successfully removed in 8 neonates. Finally, among them, 5 patients recovered and were discharged successfully, 2 patients died due to liver and kidney failure, and 1 patient died due to severe pneumonia. Patients who successfully underwent ECMO removal underwent reconstruction of the internal jugular vein and carotid artery. All patients underwent right cervical vascular colour Doppler ultrasound examination at 1 week after the operation. Four patients underwent neck MR angiography examination, and four patients underwent neck CT angiography examination. The situation of right common carotid artery patency was as follows. Colour Doppler ultrasound showed that the right common carotid artery of 5 patients was unobstructed. For these patients, CT showed mild stenosis in 3 patients, and MR showed mild stenosis in 2 patients. Doppler ultrasound and MR both showed mild stenosis in 2 patients. Doppler ultrasound showed mild stenosis in 1 patient, but CT showed moderate stenosis. The situation of right internal jugular vein patency was as follows. Colour Doppler ultrasound showed that the internal jugular veins of 6 patients were unobstructed. For these patients, CT showed mild stenosis in 3 patients, and MR showed mild stenosis in 3 patients. Doppler ultrasound and MR both showed mild stenosis in 1 patient. Doppler ultrasound showed mild stenosis in 1 patient, but CT showed moderate stenosis (Fig. 5).

The complications were as follows: 1 case had poor neck incision healing after ECMO removal. No complications, such as incisional bleeding, incisional infection, catheter-related blood infection, cannulation accidentally pulling away, vascular laceration, thrombosis, cerebral haemorrhage, cerebral infarction, or haemolysis, occurred in any of the patients.

## Discussion

ECMO is an extracorporeal life support technology that can effectively replace heart and lung function. ECMO can provide cardiopulmonary support to patients with severe heart and lung failure, thereby providing valuable time for the recovery of cardiopulmonary function [8, 9]. There are usually two types of neonatal ECMO patterns, venoarterial (VA) and venovenous (VV) ECMO. VA ECMO is established by cannulation of the right internal jugular vein and carotid artery, and VV ECMO is established by dual lumen cannulation of the right internal jugular vein. Neonates with severe respiratory failure often have severe hypoxemia, acidosis, and pulmonary hypertension, all of which can lead to heart failure. VA ECMO can provide both respiratory and cardiac support, but VV ECMO cannot provide cardiac support. In addition, for neonates, the internal jugular vein is small, and it is difficult to insert the dual lumen cannulation. Therefore, VA ECMO was the selected method for most neonates [10, 11]. VA ECMO was adopted in all neonates in our centre.

Successful cannulation is a necessary condition for the establishment of ECMO, and rapid establishment of suitable circulation access in a short time is the key to implementing ECMO [12]. At the same time, studies have found that the longer the cannulation time is, the higher the risk of infection [13]. The neonatal blood vessel wall is thin, the tissue is fragile, and the neck incision operation space is limited. To complete ECMO cannulation quickly and successfully, the operator must be familiar with the anatomical structure of neck vessels and have experience in cardiac surgery. The operators in our centre were cardiac surgeons with 10 years of experience in cardiac surgery, and the operation time of cannulation was 29 (22–40) minutes.

A stable flow rate is very important to the work of ECMO, and a good position of cannulation is the basis for a stable flow of ECMO [14, 15]. If the position of cannulation is improper, too deep, too shallow or is prone to poor drainage, it is easy to result in unstable ECMO flow. Once ECMO is stopped, the patient’s life is at risk. Frequent adjustment of ECMO cannulation would cause vascular damage and increase the risk of infection. The ECMO venous cannula has a 3 cm lateral hole. Our experience has shown that the ideal location for venous cannulation was the position of the inferior vena cava entering the right atrium. This location can allow most of the lateral holes of the venous cannulation to be located in the right atrium, which can provide stable flow. The ideal location of arterial cannulation is the innominate artery entering the aortic arch, where it would not increase left ventricular afterload. To ensure the accuracy of the cannulation position, cardiac ultrasound detection was applied after intubation, and the cannulation position was adjusted via cardiac ultrasound. Then, a bedside chest radiograph was taken to reconfirm the cannulation location. Through these methods, the intubation position of ECMO was accurate in all patients, and the ECMO flow of all patients was stable. No patients adjusted the intubation position.

Intraoperative and postoperative complications of ECMO intubation should not be ignored [16]. Shifting or ejection of ECMO tubes is a dangerous complication that can cause serious consequences. Especially for the cannula of the artery, due to the short path, intubation only needed approximately 3 cm, so cannulation was easy to remove. Stable fixation was very important, and we attached one more fixed cannula to the artery than to the vein. When the cannulation reached the correct location, we firmly fixed the cannulation of the internal jugular vein and carotid artery and closed the incisions. Outside the incision, the cannulations were sutured and fixed to the neck skin, and the external tube was fixed to the bed without tension. When moving the patients, special attention should be given to the cannulation position. Through these methods, no complications of shifting or ejection of ECMO tubes occurred.

Incisional bleeding, vascular laceration and incisional infection were common complications. It was important to be careful and use the blunt separation method to dissociate the tissue during the operation. It would not only reduce the amount of bleeding but also provide a clear position of blood vessels in the neck. When dissociating blood vessels, we should gently operate to avoid damaging the vessel wall and causing bleeding. We should also avoid excessively pulling the blood vessels; otherwise, it would result in venous contracture, which would increase the difficulty of intubation. When intubating, we should pay attention to the angle and force of the tube, and we should not intubate violently. After the cannulation entered the purse incision, the cannulation was inserted with rotating movement. At the same time, the proximal cannulation should be gently lifted to make the angle of insertion cannulation consistent with the vessel as much as possible. After successful intubation, ligating the distal end of the artery and vein, tightening the proximal end of the purse and fixing with a cannula can effectively reduce vascular bleeding at the intubation site. Intubation with ECMO is often performed at the bedside, which increases the risk of infection [17, 18]. De Rita et al. found that infection of the intubation site occurred in 15% of cases [19]. Only one case of poor neck incision healing occurred in our centre, and no incision infection or catheter-related blood infection occurred. The main reason was that the operation was performed in an intensive care unit with laminar flow equipment, and we strictly followed aseptic surgical operations. Studies have shown that the catheter-related blood infection rate can be 0% under standard intubation procedures [20].

DPolito A et al. found that the neurological complications of children with VA ECMO were higher than those with VV ECMO, and they considered the reason to be that the carotid artery was ligated [21]. Neonatal carotid arteries, jugular veins and vascular walls are thin and are difficult to anastomose and there was still the risk of obstruction or even occlusion after reanastomosis. We sought to improve the success rate of internal jugular vein and carotid artery anastomosis. The experience of our centre was to place a hose pad between the ligation line and the vessel when fixing the proximal end of the cannulation and ligaturing the distal end of the jugular vein and carotid artery. In this way, it was not only easier to remove the ligation line but also did not damage the blood vessels. We removed the purse line of 5–0 prolene, and then we used the 8–0 prolene line to anastomose the jugular vein and carotid artery. Noninvasive forceps were used to protect the vasculature during anastomosis. In our study, 8 patients underwent successful reconstruction of the internal jugular vein and carotid artery. Arterial blood flow was unobstructed in 5 patients, mild stenosis in 2 patients, and moderate stenosis in 1 patient. Venous blood flow was unobstructed in 6 patients, mild stenosis in 1 patient, and moderate stenosis in 1 patient.

This study had some limitations. First, this study was a retrospective study with the absence of a control group, which would cause biases and confounding factors. Second, the study's sample size was limited to 12 neonates, so the results may not be representative of the general neonatal population. Third, the study lacked long-term outcomes. The long-term follow-up data are currently in progress, and we will report them in the future.

## Conclusion

Cannulation of the internal jugular vein and carotid artery can quickly establish effective ECMO access for neonates with severe respiratory failure. Careful, skilled and delicate operation was essential. In addition, during the cannulation process, we should pay special attention to the position of cannulation, firm fixation and strict aseptic operation.

## Data Availability

The datasets of the current study are available from the corresponding author upon reasonable request.

## Abbreviations

ECMO: Extracorporeal membrane oxygenation
VA: Venoarterial
VV: Venovenous
